# Colloidal Solutions as Advanced Coatings for Active Packaging Development: Focus on PLA Systems

**DOI:** 10.3390/polym15020273

**Published:** 2023-01-05

**Authors:** Athira John, Klementina Pušnik Črešnar, Dimitrios N. Bikiaris, Lidija Fras Zemljič

**Affiliations:** 1 Laboratory for Characterization and Processing of Polymer Materials, Faculty of Mechanical Engineering, University of Maribor, 2000 Maribor, Slovenia; 2 Laboratory of Polymer Chemistry and Technology, Department of Chemistry, Aristotle University of Thessaloniki, GR-541 24 Thessaloniki, Greece

**Keywords:** food packaging, active packaging, colloids, PLA

## Abstract

Due to rising consumer demand the food packaging industry is turning increasingly to packaging materials that offer active functions. This is achieved by incorporating active compounds into the basic packaging materials. However, it is currently believed that adding active compounds as a coating over the base packaging material is more beneficial than adding them in bulk or in pouches, as this helps to maintain the physicochemical properties of the base material along with higher efficiency at the interface with the food. Colloidal systems have the potential to be used as active coatings, while the application of coatings in the form of colloidal dispersions allows for prolonged and controlled release of the active ingredient and uniform distribution, due to their colloidal/nano size and large surface area ratio. The objective of this review is to analyse some of the different colloidal solutions previously used in the literature as coatings for active food packaging and their advantages. The focus is on natural bio-based substances and packaging materials such as PLA, due to consumer awareness and environmental and regulatory issues. The antiviral concept through the surface is also discussed briefly, as it is an important strategy in the context of the current pandemic crisis and cross-infection prevention.

## 1. Introduction

The Consumer demand and government legislation around the world are key drivers of the sustainable packaging agenda. Environmental and health awareness among a growing number of consumers is driving demand for sustainability and safety, as well as reducing the impact of packaging on the environment [1]. At the same time, sustainability programmes are increasingly seen as a source of innovation that can help differentiate companies by appealing to consumer awareness and serving as a platform for new products and market development. Therefore, progress needs to be made in reducing the environmental footprint (in terms of biodegradability and sourcing of packaging raw materials) and cost of packaging, as well as improving safety [2]. According to recent surveys, plastic packaging waste generated in Europe is expected to increase from 23 million tons to 92 million tons by 2050, which is an alarming state of affairs. The main problem for the environment is that plastic waste breaks down to microscopic amounts and degrades over hundreds, or even thousands of years. It is eaten by wildlife, absorbs toxic compounds and transports them through our soils and waterways, forming intricate webs that take a heavy toll on ocean biodiversity. This is a serious problem [3].

**Figure 1:**
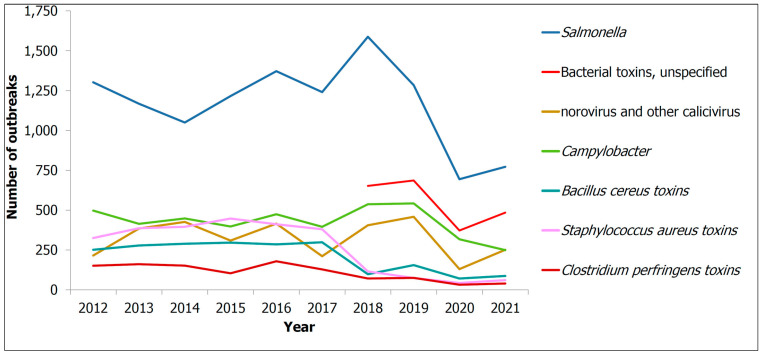
Number of foodborne diseases due to foodborne outbreaks in 2010–2021 Reprinted with permission from ref. [5].

**Figure 2:**
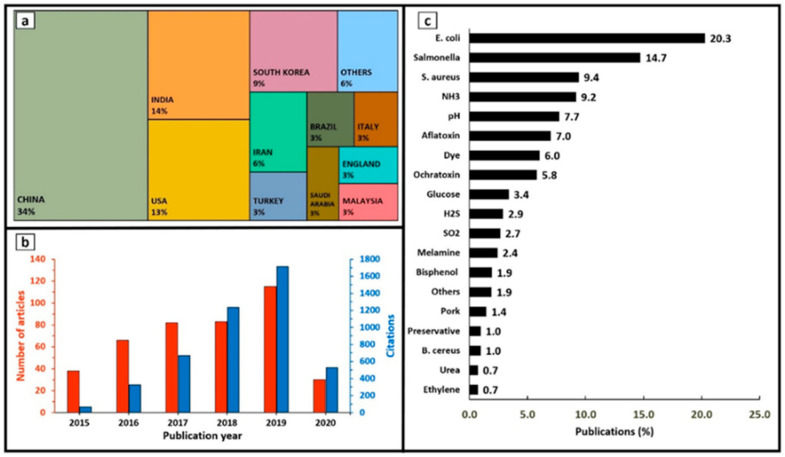
An overview of publications on food spoilage during 2015–2020 in relevance to (**a**) countries, (**b**) publication year, and (**c**) analytes. Reprinted with permission from ref. [9].

On the other hand, this decade has seen a startling increase in the number of illnesses, hospitalisations and deaths, due to foodborne infections in the EU. In 2018, 5098 foodborne epidemics with half a thousand cases of illness, about 5000 hospitalisations and 40 deaths, and it is also worth noting that the number was 5000 cases lower in 2017 [4]. Figure 1 shows the number of illnesses due to foodborne and waterborne outbreaks in the EU from 2010 to 2021, [5] with pathogens such as *Campylobacter, Salmonella, Bacillus cerus, Staphylococcus aureus,* etc. [6]. Inexplicably, according to an estimate by Holland et al., foodborne illnesses caused by *Campylobacter* and *Salmonella* were responsible for 33 and 21 deaths, respectively, in the UK alone in 2019. Undoubtedly, improper storage, including inadequate packaging, is one of the major causes of this food contamination [7]. Figure 2 shows a summary of the articles published on food spoilage in the last decade. From Figure 2, it can be seen that food spoilage and its causes have attracted a lot of interest over the years, and it represents a big problem. In addition, bacteria such as *E. coli* and *Salmonella* are the main contributors to food spoilage, prompting the development of antibacterial packaging [8].

**Figure 3:**
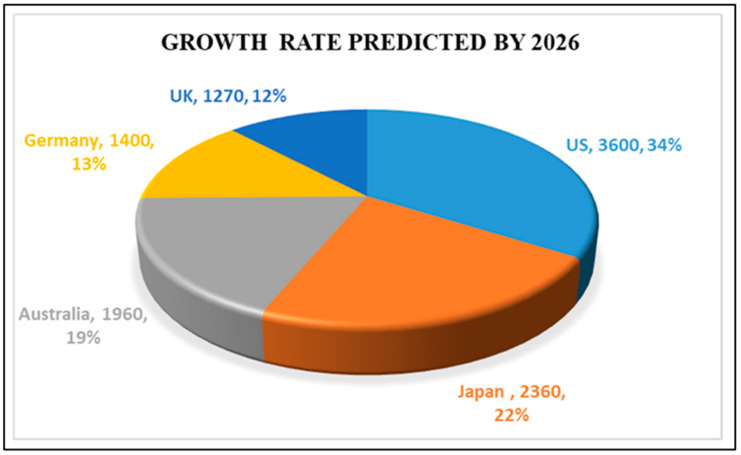
Future of global markets for active packaging by the year 2026 Reprinted with permission from ref [16].

Active systems can be used successfully to extend the shelf life of processed foods, and can be divided into adsorbent and releasing systems (e.g., oxygen scavengers, ethylene scavengers, liquid and moisture absorbers, taste and odour absorbers or releasers, antimicrobials, etc [10,11,12,13,14]. On the other hand, smart packaging can monitor the condition of the packaged food or the environment by providing information on various factors during transportation and storage, including time-temperature indicators, gas detectors (volatile organic compounds (VOCs) and gas molecules like H_2_, CO, NO_2_, O_2_, NH_3_, etc.) and freshness and/or ripeness indicators. Although active packaging attracted interest in Japan as early as the 1970s, it gained significant attention in European markets in the 1990s. The future of global active packaging markets through 2026 is shown in Figure 3 [15]. In light of these developments, eco-innovative and functional food packaging materials are fast becoming a strategic target for the European food sector and market.

To meet the demands of the market, new innovative, biodegradable and functional materials that combat pathogens, reduce spoilage and waste, optimise process efficiency, reduce the need for chemical preservatives, improve food functionality, and enhance the nutritional and sensory properties of food (in response to the demands of different consumer niches and markets) must be developed and brought to market [10].

In the last two decades, many excellent and detailed reports have been published in the literature on active packaging, coatings for active packaging, biodegradable packaging, etc., [17,18,19,20]. However, to our knowledge, there is a shortage of exclusive reviews on colloidal solutions as coatings for food packaging. Colloids are particles in the size range of 1 to 1000 nm, which provide a high surface area of interaction, and, thus, as preservatives, improve the freshness and shelf life of food efficiently while monitoring storage conditions from the point of production to consumption by the end consumer [21]. Due to their colloidal size, a smaller amount of active agents is required, which also reduces the cost. Moreover, encapsulation in these particles allows controlled release of active ingredients to be in accordance with migration requirements. This work will highlight the advantages of colloids as natural coatings, and clarify why colloidal formulations are preferred over conventional ones. It will also discuss the state-of-the-art in existing colloidal coatings, focusing on different structural forms of colloids. Furthermore, because of their adaptability and potential to help create biodegradable sustainable packaging, which is now a necessity in the modern world, poly lactic acid (PLA) films used in active packaging will be discussed thoroughly in each section [22,23].

Finally, an additional attempt is made to discuss the challenges and efforts to achieve SARS-CoV-2 inhibitory properties to counter the current pandemic scenario. SARS-CoV-2 viruses can remain active on packaging surfaces for up to several days, and therefore pose a high risk of cross-infection from packaging surfaces in stores. If a packaging surface contaminated with the virus is touched by the next user, the virus will be transmitted to him/her [24]. Therefore, developing both antimicrobial and antiviral coatings in one stable concept is a major challenge nowadays [10,25,26].

## 2. Basic Packaging Materials

It is surprising that, while packaging serves to prevent food spoilage and thus make the earth waste-free, the packaging itself results in a huge pile of waste that leaves the earth as an enormous amount of waste with a high CO_2_ emission rate and poses a threat to aquatic and terrestrial life [27,28,29]. After the era of glass, metal and paper, the food packaging industry saw the ground-breaking emergence of plastics. Due to low production costs, durability and other unique features, companies continue to rely on plastic packaging. It has been noted that plastic packaging is the largest market for plastics, accounting for nearly half of the plastic waste produced worldwide. Polypropylene (PP), low-density polyethylene (LDPE), polyvinyl chloride (PVC), polystyrene (PS), high-density polyethylene (HDPE), and polyethylene terephthalate (PET) account for the majority of this contribution [30]. The global generation of plastic waste has more than doubled from 2000 to 2019, reaching 353 million tonnes. Approximately two-thirds of plastic waste comes from plastics with a lifespan of less than five years, including 40% from packaging, 12% from consumer products, and 11% from clothing and textiles. Single-use plastic packaging is a major component of the millions of tonnes of plastic waste that escape collection systems and end up in waterways each year [29].

Although packaging has an important function in the global economy, producer responsibility policies remain scarce at the national and international levels. The findings show that the transition to sustainable packaging in the food and beverage sector has been slow and uneven. Most corporate sustainability reports do not address plastic pollution [31]. In addition, companies tend to report on collection and recycling rather than sustainable packaging solutions aimed at systemic change. Unfortunately, today’s widely used plastics, such as PS, PP, PE, PV and PET, are all made from petroleum. Their production is associated with gas emissions, hazardous chemical residues, high energy consumption and high-water consumption, which affects CO_2_ emissions. Their potential end of life is incineration leading to greenhouse gas emissions, accumulation in landfills, and degradation into microplastics causing imbalances in the food chain. Also, a significant amount of plastic waste accumulates in the oceans, which is detrimental to aquatic animals [27]. The accumulation of microplastics in the aquatic ecosystem also leads more broadly to ecosystem disruption. The European Parliament called for urgent action to reduce aquatic pollution, and the EU is proposing several new pieces of legislation against plastic pollution [32].

Although synthetic plastics have superior mechanical and barrier properties, they are not biodegradable and are the biggest environmental threat today, so there is growing concern about the environmental hazards they pose. According to the United Nations (UN), food production, packaging and distribution account for one-third of greenhouse gas emissions and up to 80% of biodiversity loss on earth, which is expected to increase by up to 40% by 2050 if no action is taken. Therefore, appropriate measures for sustainability are needed, and the food packaging sector is nowadays in the maelstrom of developing new environmentally friendly alternatives [33]. With the development of new materials, this sector plays an important role in the development of novel sustainable packaging solutions, and can help to switch to a greener earth with more sustainable alternatives. This quest led to the call for more environmentally friendly alternativebioplastics.

However, bioplastic is an umbrella term that needs to be defined precisely, so that consumers do not get the wrong idea to put anything labelled “bioplastic” somewhere and believe that it will decompose immediately into humus. Pursuant to the EU Bioplastics, the industry association, bioplastics are plastics that are either bio-based (of biological origin) or biodegradable, or both. All materials that are partially or fully derived from biomass are biobased, but they do not necessarily have to be biodegradable. Anything is biodegradable if it undergoes degradation by microorganisms in the environment and is converted into natural substances [34]. Apparently, the third category of bioplastics may seem ideal, but all of them contribute in some way to a greener earth, for example, by reducing the carbon footprint and greenhouse gas emissions. Undeniably, fossil fuel savings are also a boon [35].

Yet again, biodegradability is confused with compostability, the difference between which is characterised by a very narrow line where compostable plastics degrade to non-toxic residues within a certain time frame, while the former has no time frame. Technically, compostable plastics must degrade within 180 days, leaving no visible or toxic residue, while biodegradable plastics might take longer and can leave toxins behind. Compostability can be classified further as home compostable or industrial compostable. The former are plastics that compost at much lower temperatures and over a long period of time, while the latter require a highly controlled environment with specific temperatures and conditions for composting [36].

**Figure 4:**
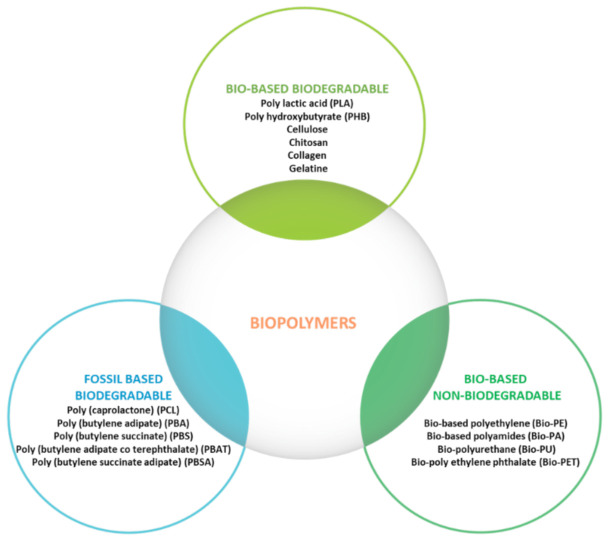
Biopolymer classification.

It is obvious that there are many polymers, including biopolymers, that can be used to produce biodegradable basic packaging material, but unfortunately not all of them can offer promising physicochemical and mechanical properties for actual use, which is why most of them are used as edible films or functional agents as fillers or coatings for basic packaging materials (Figure 4) [37].

**Figure 5:**
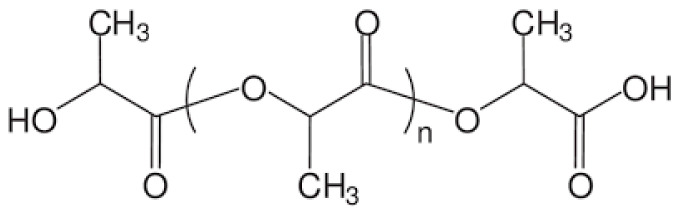
PLA chemical structure.

Polylactic acid (PLA) and its derivatives and composites are the most promising biopolymer emerging as a replacement for low-density (LDPE) and high-density polyethylene (HDPE), polystyrene (PS), and so on. A large part of the review is therefore devoted to this material [38,39]. PLA belongs to the class of aliphatic polyesters, and can be prepared by fermentation of carbohydrates or by chemical synthesis of lactic acid monomer (LA) (Figure 5) [39]. PLA is obtained either by direct polycondensation of L- and/or D-lactic acid monomers, or by ring-opening polymerisation of lactide monomers. PLA is a semi-crystalline thermoplastic with a melting point of 150 °C to 160 °C and a glass transition temperature of about 60 °C [40]. PLA can be processed by extrusion, injection moulding, casting, blown filming, thermoforming and filament spinning [40,41].

PLA meets most of the conditions for food packaging, because it is transparent. In addition, PLA has high mechanical strength and low toxicity [42]. However, PLA also has some disadvantages, such as low flexibility, poor crystallisation behaviour, and poor barrier properties.Moisture and oxygen can penetrate it more easily than other plastics. It is stiff and quite brittle at room temperature. It also does not compost fast enough for industrial composting facilities. The residues are not compost. They do not improve soil quality. Therefore, intensive research is being conducted to improve these properties of PLA by mixing it with compounds that have better properties. For instance, Pušnik et al. studied the changes in surface properties, antibacterial activity, and mechanical properties of PLA films by adding metal-based nanoparticles, namely, Ag, ZnO, and TiO_2_ [38]. In another work by the same group, the improvement of properties was investigated by the addition of wood fibres, kraft lignin and tannin [43]. Giulia Fredi et al. demonstrated successfully that mixing PLA with different poly(alkylene furanoates) would improve the mechanical properties and gas barrier properties, making it more suitable for packaging [44]. It has also been reviewed that blending PLA with a triester of glycerol and acetic acid, as a plasticiser, blending with ZnO nanoparticles as a reinforcing material, and blending with other biopolymers such as PBAT (polybutylene adipate terephthalate) in optimal amounts improved the mechanical properties, including the tensile strength, elastic modulus, and elongation at break of PLA [45].

## 3. Fundamentals of Active Packaging

Active packaging is an evolving technology used to retain the quality of food. According to European Regulation, active materials are those components that are introduced intentionally into/onto the packaging to extend the shelf life or maintain or improve the condition of the packaged food, and are purportedly designed to release or absorb substances into or out of the packaged food or the environment surrounding the food [46]. They can also act without being released on the surface if they come into contact with the food at the interface. Since this technology omits the stage of adding anything directly into the food, it is convenient and satisfactory from both the manufacturer’s and consumer’s perspectives. Active packaging films have been employed successfully in the packaging of food products such as meat and fish products, dairy products, vegetables, bread and bakery products, etc., [47].

An appropriate understanding of the spoilage mechanism of food is adequate for the targeted application of active packaging. Food spoilage is any change in food quality that makes it less consumable. Colour, smell, flavour and texture are the identifiers for food deterioration. Spoilage results from a variety of mechanisms, ranging from physical to chemical to biochemical, or even microbiological. Spoilage produced by one mechanism can trigger another, hence, these mechanisms are not always mutually exclusive [48].

Physical food spoilage is described as food that has undergone physical alteration or instability; it includes things like moisture gain or loss, moisture movement between distinct components, and physical separation of ingredients or components. The most contributing physical element to the rotting of fruits and vegetables is temperature. The right temperature range can promote post-harvest vitality and slow ripening.

Food undergoes chemical and biological reactions inherently, which provide unappealing sensory responses in food products. Chemical and biological reactions in food can cause quality changes due to variables such as microbial growth/metabolism toxic compounds, the oxidation of lipids and pigments in fat, leading to unpleasant odours and colours. Oxidation is a typical chemical reaction that causes food degradation by converting amino acids into organic acid and ammonia. Putrefaction is another type of anaerobic reaction that can occur in food, in which amino acids degrade to a mixture of amines, organic acids, and foul-smelling sulphur compounds such mercaptans and hydrogen sulphide. The Maillard reaction, also known as enzymatic browning, occurs in the amino group of proteins, or amino acids found in foodstuffs. Chemical spoilage is linked to microbiological spoilage [49].

Microbial spoilage is the most common spoilage found in food. The colonisation of exposed food by bacteria and yeasts may go unnoticed for a longer period, since they are too undetectable by naked sight. Food spoilage microorganisms can colonise food in various ways according to the complexity of food and the interconnection of the elements that contribute to it. The most frequent causes of food spoilage in lipid-containing foods, such as meat and fish, are bacteria, those who prevail, produce metabolites, which change the organoleptic properties of meat and render it unfit for consumption [50].

There are a variety of active packaging systems with different active functions that are applied, depending on the spoilage mechanism. They are classified broadly into releasing and non-releasing systems (Table 1) [9,51]. A variety of active agents with various functionalities may be incorporated into the packaging material to provide the desired functions, such as absorbing/scavenging properties (e.g., oxygen, carbon dioxide, ethylene, moisture, flavours, and UV light); releasing/emitting properties (e.g., ethanol, carbon dioxide, antioxidants, preservatives, sulphur dioxide, and flavours); and antimicrobial agents that can act stably at the surface interface, or be released in a controlled manner [3]. Achieving multifunctionality in one hand is a big issue today.

**Figure 6:**
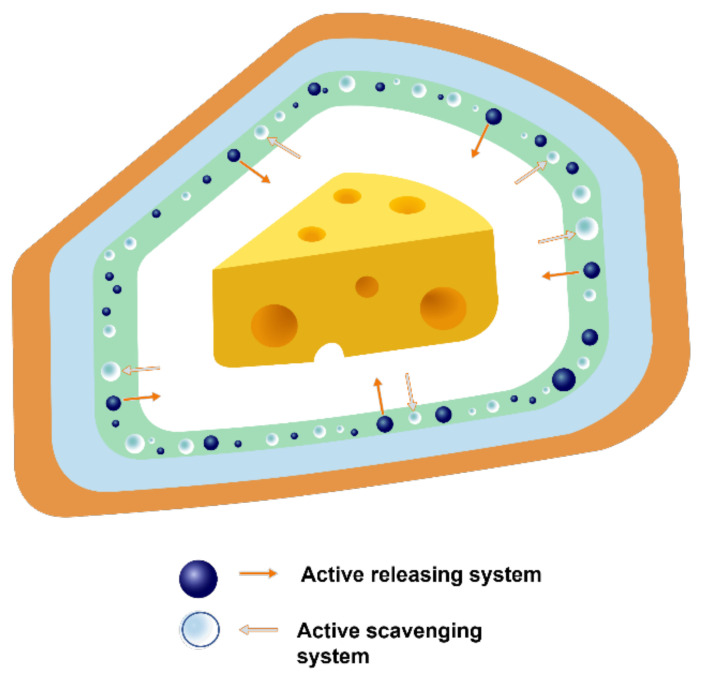
Active packaging system.

**Figure 7:**
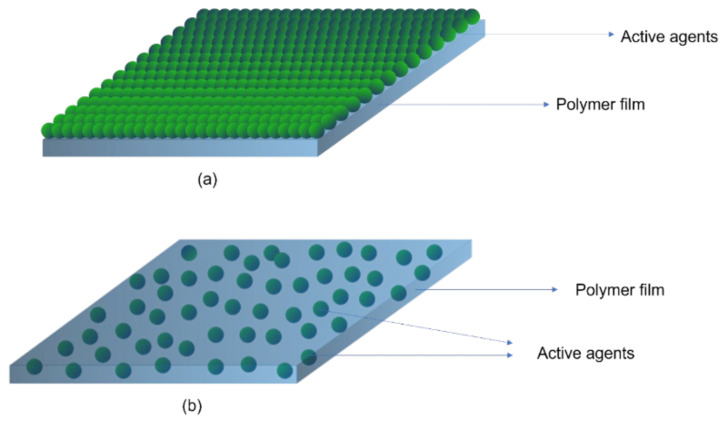
Schematic representation of active agents coated (**a**) and integrated into bulk of polymer films (**b**).

A schematic diagram of active packaging is shown in Figure 6. The positioning of the active agent in a package is another point of discussion. Additives can be incorporated into the material either by direct addition into the bulk of the material, e.g., extrusion, blow moulding, injection-blow moulding, etc., or by application as a coating to the surface (Figure 7). Thus, active packaging often involves placing compounds into different formats, such as labels, packaging films, labels, and coatings [61].

## 4. Coatings

Although bulk incorporation, such as extrusion and blending, are high-temperature processes and are used extensively for active packaging, there is the serious limitation that the vast majority of active agents, especially natural ones, are thermally unstable, and therefore could be degraded easily during processing [61]. By coating on the surface heat-sensitive agents like enzymes, biopolymers such as polysaccharides and volatile active ingredients such as sulphur dioxide etc. can be protected from the possibility of exposure to high temperatures or shear forces, and therefore can retain their functionalities. In addition, placing the active agents on the surface is considered advantageous because it preserves the inherent properties of the bulk material as such. Better action at the interface, a larger contact area with the food, possible controlled release, etc. are other advantages [62].

**Figure 8:**
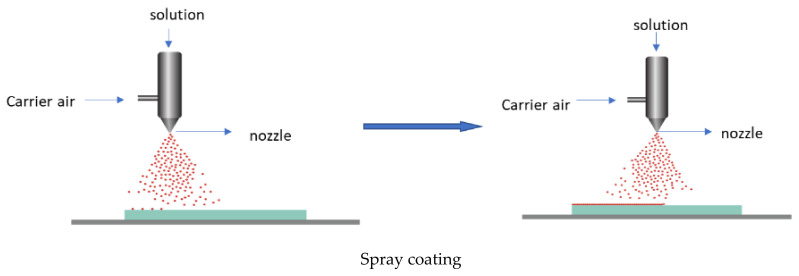

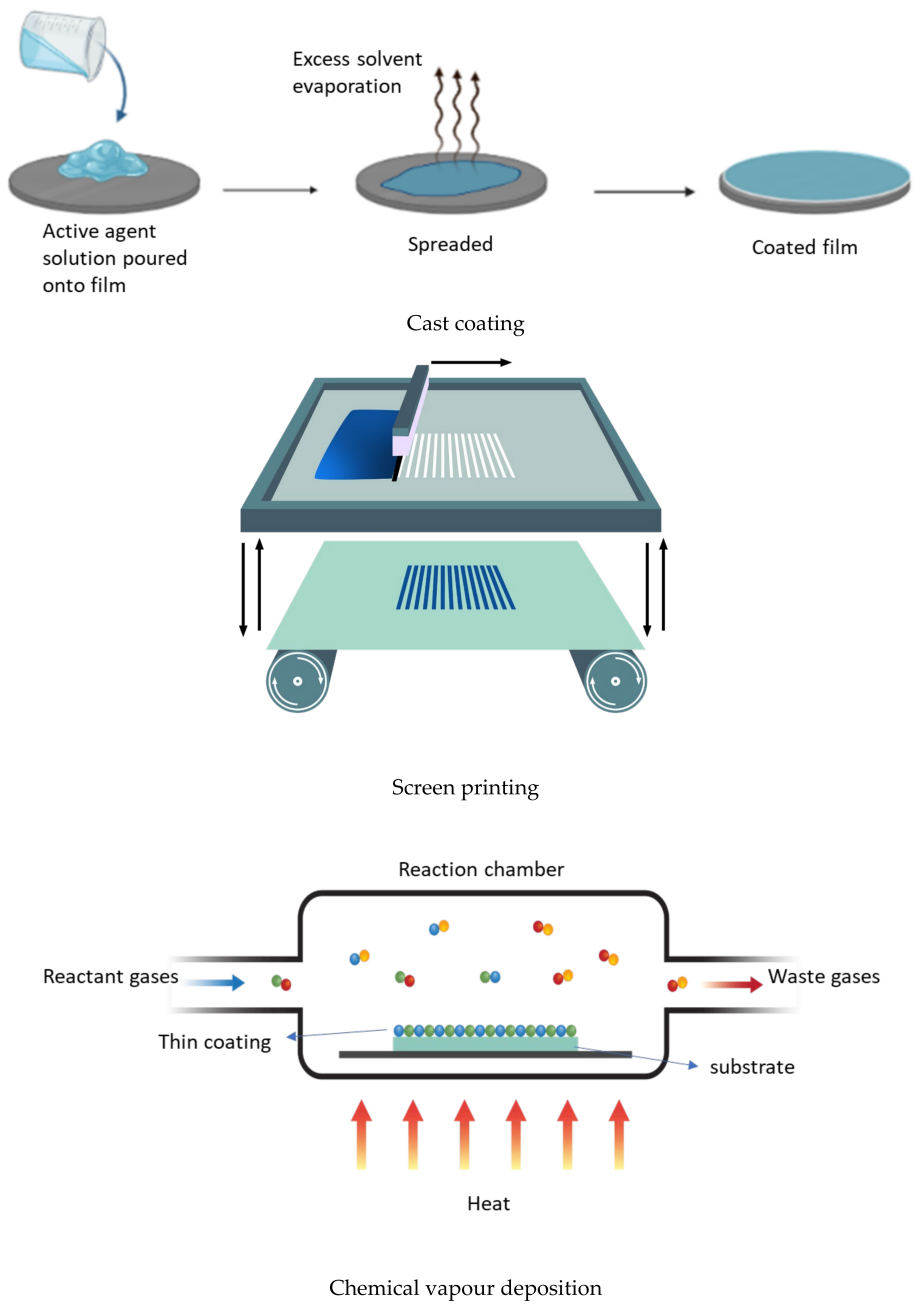

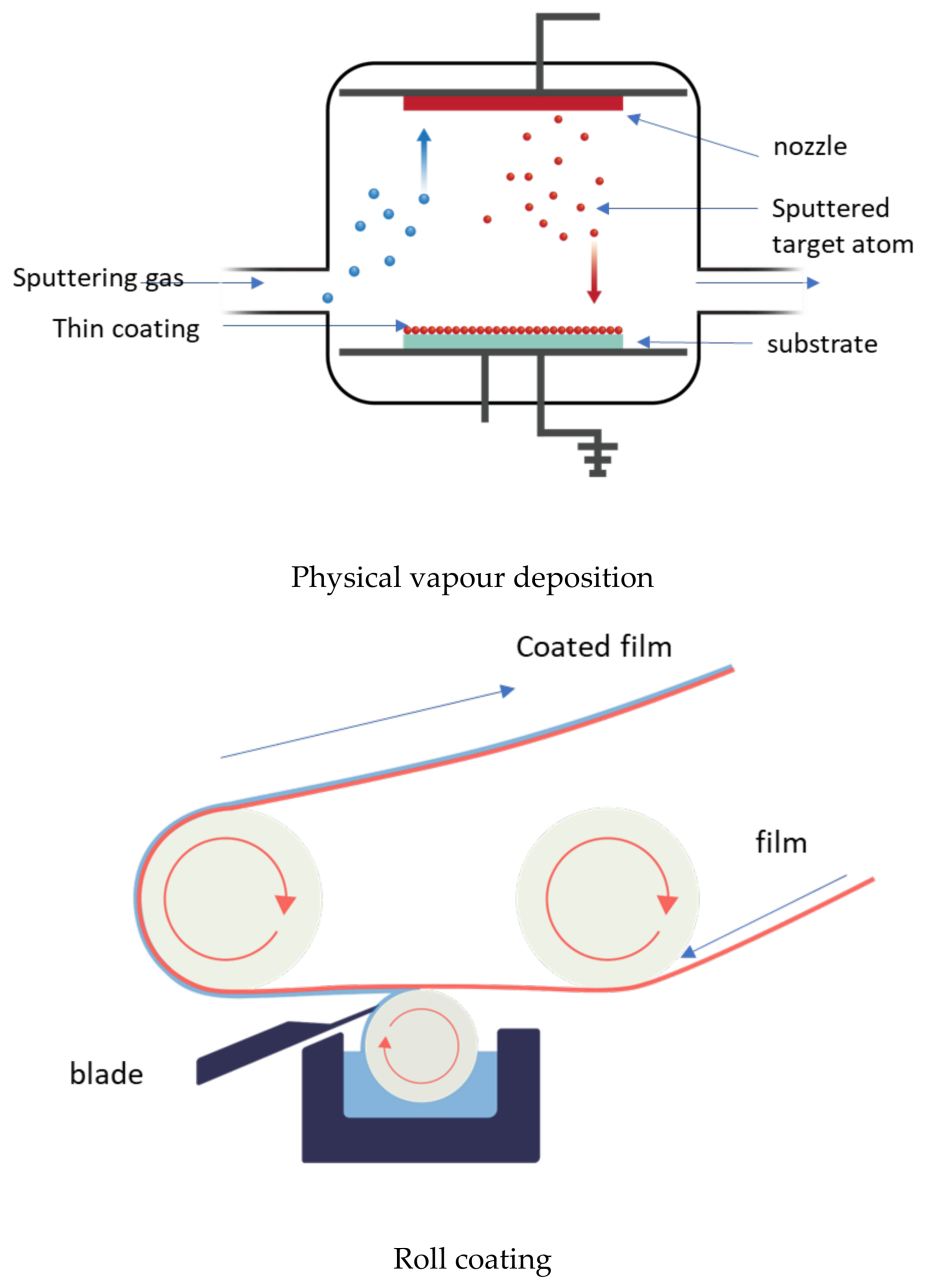
Some of the coating technologies.

Technically, a coating is the deposition of a continuous layer of a material to a substrate, in our case a polymer-based film. These coatings serve as a functional layer, or a layer for embedding the active agents. There are several established methods, such as spraying, electrospraying, dipping, cast coating, chemical vapour deposition, physical vapour deposition, roll-to-roll, and printing (screen printing) used for coating (Figure 8) [63]. Spray coating is the process in which the active agents (usually melted) are forced through a nozzle using high pressure (sprayed) and deposited onto the surface to form a coating. Dip coating is a process by which the film is submerged in the coating mixture and taken out and dried. Cast coating is when the active agents are dissolved in a suitable solvent and poured onto the surface of the film and allowing the solvent to evaporate. Chemical vapour deposition (CVD) is the technique in which the active substances that are in the vapour phase are condensed to generate a solid thin layer on the films [64]. Physical vapour deposition (PVD) involves the deposition of a thin, solid layer on the film by the condensation of a vapourised form of the solid substance, i.e., in CVD coating the material is in gaseous form, and the atoms moving around will react with the film’s surface, while, in PVD, it is in solid form, and does not react chemically with the substrate. Roll coating is a technique which uses a series of rollers to apply the coating liquid on the substrate. Screen printing is a process in which the coating substance is forced through a mesh screen onto a surface of the film [65]. The resulting films will possess the targeted functionality. The coating method is chosen depending on the physical and chemical nature of the substrate as a coating, and the physical, chemical, and structural form of the adsorbed coating. The coating can be either single-layer or multilayer, using the layer-to-layer technique [66,67].

**Figure 9:**
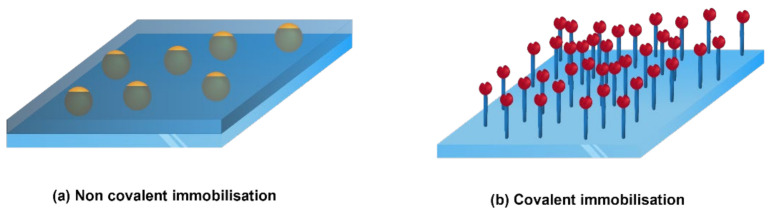
Surface immobilisation methods.

**Figure 10:**
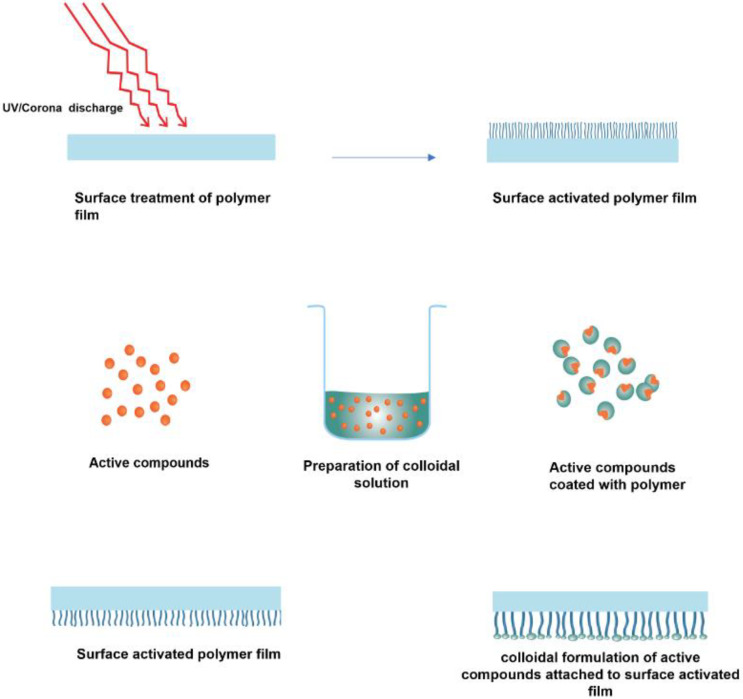
Schematic representation of covalent grafting of colloidal coatings.

It also depends on whether the active agents are intended to migrate to the food or not [61]. The active compounds can move from the package by diffusion, evaporation, or remain fixed through chemical immobilisation. The surface immobilisation can be either through a non-covalent bond or through a covalent bond (Figure 9). In covalent immobilisation, the active compounds do not migrate into the food, whereas in non-covalent immobilisation (physical interactions), the active ingredients migrate (in an uncontrolled or controlled manner) into the food or the headspace of the food to deliver the assigned activity (antibacterial, antioxidant, etc) [68]. Covalent grafting of bioactive compounds onto inert polymeric surfaces often occurs after surface activation, e.g., by various plasma activations or hydrolysis [34]. (Figure 10).

Ideal packaging material can thus be defined as a biodegradable active material with active agents on the surface that does not release unwanted contaminants into the food [68]. For films where the agents are intended to migrate into the food, or vice versa, this must occur in a controlled manner [69]. For this reason, the conditions for adsorption of the coating must be optimised for each specific technology (pH, ionic strength, concentration, temperature, etc.) to understand the manipulation of agent binding and predict a controlled release manner. Even if migration is intentional for some active ingredient films, it must, in any case, comply with the Regulations of agencies such as the Food and Drug Administration (USA) and the European Food Safety Authority (European Union), which provide the legal basis for their precise use, safety and marketing [70]. For example, according to Article 11 of Regulation (EC), No. 882/2004 of the European Parliament and of the Council, the migration of compounds not in direct contact with food must not exceed 0.01 mg/kg. Furthermore, it is essential that food packaging should necessarily be carrying information on the permitted use, and other relevant information such as the name and maximum quantity of substances released by the active component while being marketed [71].

While it has already been suggested that the perfect active package has the active components on its surface, it is emphasised that a thin homogeneous layer or multiple layers of coating containing the active ingredient are key to this task. Therefore, in addition to the desorption/migration study, the morphology of the surface must also be considered. Especially, the thickness and roughness of the coatings is important [72]. In addition, these coatings must have high transparency, because transparent packaging allows consumers to assess the quality and freshness of food products better. When customers can see through the packaging, they get the assurance that they will buy the product [65]. Properly manufactured, transparent packaging can help prolong the freshness of food, extend its shelf life, reduce waste, and address consumers’ growing environmental and health awareness [73].

## 5. Coatings as Colloidal Formulation

Colloids (also called colloidal solutions or colloidal systems) are mixtures in which microscopically dispersed, insoluble particles of one substance are suspended in another substance. The size of the suspended particles in a colloid can range from 1 to 1000 nm (10^−9^ m). A colloidal system consists of two separate phases: a dispersed phase (or internal phase) and a continuous phase (or dispersion medium). A colloidal system can be solid, liquid, or gas. The substance that is dispersed is called the dispersed phase, and is suspended in the continuous phase [74]. Since the dimensions of most “colloidal systems” also fall into the nanoscale and/or are formed by the association of nanosized particles with various colloidal interactions, nanotechnology and nanoparticles are also important in this regard.

**Figure 11:**
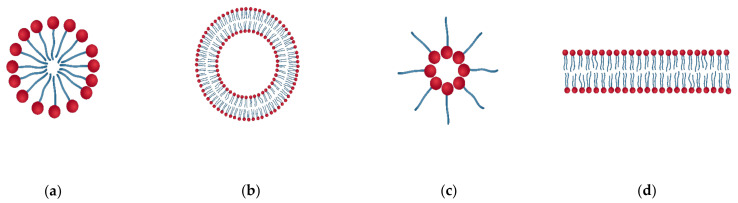
Types of associated colloids (**a**) Micelle (**b**) Bilayer (**c**) Reverse micelle (**d**) Vesicle.

Based on the nature and structure of the dispersed phase particles, they are divided into multimolecular, macromolecular, and associated colloids. The multimolecular colloids consist of atoms or small molecules (with a size of ≥1 nm) aggregated together, such as metal and metal oxide nanoparticles, that form aggregates and are deposited on polymer films. Macromolecular colloids are individual macromolecules like polymers, that are large enough to be considered as colloids. This type of colloid is made up of high molecular weight compounds [75]. Colloidal particles can also be prepared from macromolecules by simple methods such as ionic gelation, precipitation, and coacervation [21]. Proteins, polysaccharides, starch, enzymes, etc. are some naturally occurring macromolecular colloids. The third class of colloids are associated colloids. They are formed by particles that behave like strong electrolytes at low concentrations, but like colloids at high concentrations [22]. They are formed by the association of compounds such as long-chain fatty acids, quaternary ammonium compounds, etc. There are different types of association colloids, such as surfactant micelles, vesicles, bilayers, reverse micelles, etc. (Figure 11).

Liposomes and niosomes are colloidal association of amphiphilic lipids that organise spontaneously into bilayered vesicles, and that are suitable for hydrophilic and hydrophobic compounds. A colloidal system containing two immiscible liquids, a lipid-based hydrophobic material dispersed in the water phase in the form of droplets is called a nanoemulsion, which is of immense importance for active packaging based on colloidal coatings [76].

The main advantage of colloids is that they increase the circulatory volume due to their immense volume size. Due to their specific surface area, they are very efficient and active as a coating, increasing both the absorption rate on the surface and the activity of the coated surface. Colloidal systems are known to be ideal systems for controlled delivery of active ingredients [22].

- The main advantages of using colloidal solutions as coatings for active packaging films are:
- The release of the active agents can be controlled by the strength of the ions, the ambient temperature and the pH, which allows for sustained and controlled release
- Homogeneous distribution and efficient thickness of the active agent can be ensured, especially when using macromolecular solutions coated in extended conformation
- Higher stability
- Larger surface area ratio, reducing the unstable release caused by the accumulation of the active ingredient, thus improving its bioavailability.
- Low consumption due to its efficiency (especially at the nanoscale), and thus economic and environmental impact [77].

**Figure 12:**
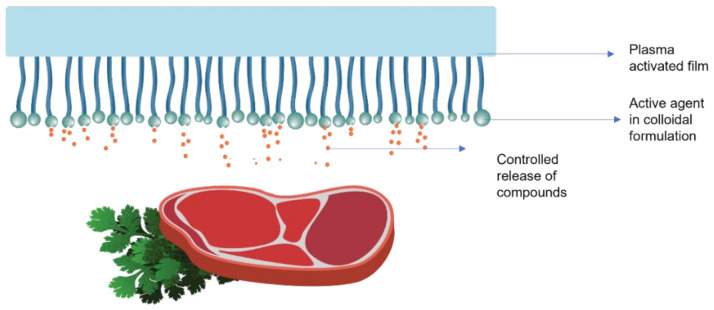
Schematic representation action of coatings as a colloidal formulation in active food packaging.

Roll-to-roll, spraying, and dipping techniques are generally used for these coatings on the material’s surface. The steps followed extensively in the application of coatings in the form of colloidal formulations are already shown in Figure 10, and a typical release of compounds from colloidal coatings is shown in Figure 12.

### 5.1. Multimolecular Colloidal Coatings

Certain inorganic components, such as TiO_2_, ZnO, silver nanoparticles, etc., are approved for use in food, and are considered food contact materials by the American Food and Drug Administration (FDA). They are generally considered safe (GRAS) [78]. These nanoparticles aggregate together to form the multimolecular colloidal formulation, and are coated successfully to the surface of the material [23,79]. Some of them are known for their antibacterial properties, while others improve the gas barrier and mechanical properties when coated onto the material’s surface. TiO_2_ is a non-toxic species capable of generating hydroxyl radicals and reactive oxygen species that inactivate microbes by destroying the polyunsaturated phospholipid components of the cell membrane [80]. The antimicrobial activity of colloidal silver nanoparticles prepared by the sol-gel method was studied by N. Lkhagvajav and coworkers. The antimicrobial activity of this system is attributed to the silver atoms released at the interface, which interact with and break the cell wall of the bacteria [81]. A suspension of Ag nanoparticles was applied to the outer polyamide layer (PA-6) of a multilayer packaging film made of co-extruded PA6—PE film by Valdes et al. The suspensions in a water-ethylene glycol solution with each of the two different anhydrides, itaconic anhydride and maleic anhydride, were carried out using a sonicator. The resultant suspensions were coated on the film by ultrasonic sputtering and spraying. Ultrasonic sputtering produced the smallest average silver particle size compared to spraying, which had a significant effect on the antifungal mechanical and optical properties of the packaging. The ultrasonic deposition method and itaconic anhydride also formed the best nanoparticle dispersion and the lowest agglomeration. The smaller the particle size and the lower the agglomeration, the better the properties were [82]. Similarly, the antibacterial effect of various thermoplastics, such as medium density polyethylene (MDPE), polystyrene (PS), polyethylene terephthalate (PET), and polyvinyl chloride (PVC) containing nanosilver colloids, was investigated by spray coating, and direct blending was studied by Pongnop et al. under a variety of test conditions. It was found that spray coating was much more effective than blending [83].

In addition, ZnO-coated PVC films developed by Li et al. exhibited a decrease in the water vapour transmission rate of the film from 128 to 85 g/m^2^/24 h. It was observed that the ZnO nanoparticles were dispersed uniformly on the surface and hardly formed agglomerates. The ZnO nanoparticle dispersion stabilised by PEG-400 was applied to the poly(vinyl chloride) (PVC) film, and the resulting film was found to have good potential to be used as an active coating system for food packaging, because it has excellent barrier properties and antibacterial properties due to the nanosize of the ZnO [84].

Woo Kim et al. developed another packaging film coated with inorganic materials; the biaxially oriented polypropylene film (BOPP) was coated with hybrid sols of polyvinyl alcohol (PVA) and inorganic silicate by the spin coating method. The PVA solution was mixed with partially hydrolysed silica sol, to obtain PVA/SiO_2_ hybrid coating solutions with different compositions, and was spin-coated onto pre-treated BOPP (biaxially oriented polypropylene) films with a thickness of 40 µm under the conditions of a rotation speed of 6000 rpm and a coating time of 30 s. It was found that the oxygen barrier property of the PVA/SiO_2_ hybrid coated film was increased by 50 times compared to pure BOPP [85].

Despite its advantages, such as excellent mechanical properties, transparency, and commercial availability, polylactic acid has a limitation in the use of gas barrier films for food packaging, because it has relatively low resistance to the permeation of oxygen and water vapour compared to conventional materials. The endowment of active functions would make PLA films an exceptional material for food packaging. Literature is listed on the use of multimolecular colloidal coatings to improve the properties of PLA films for food packaging.

Valerini et al. applied aluminium-doped zinc oxide successfully as a coating to extruded PLA films for food packaging using magnetron sputtering. The resulting films exhibited antibacterial and UV-blocking properties and were transparent, making them well suited for active food packaging [86]. Zhang et al. coated PLA films with ZnO nanoparticles for antimicrobial food packaging, and 0.5 wt% of the nanoparticles were effective in inactivating *E. coli* [87].

Marra et al. used 5% ZnO particles and stearic acid coated ZnO particles (ZnOc) as a coating for PLA. Homogeneous dispersion and distribution of ZnOc particles and ZnO on the PLA matrix occurred, resulting in improved tensile properties. Both the ZnO and ZnOc particles shielded the UV radiation on the PLA. The coated particles also affected the thermostability. In addition, PLA/ZnO showed a significant oxygen barrier and enhanced antibacterial activity against *E. coli* [88].

Pedron et al. coated a commercial PLA film with tungsten oxide (WO_x_) by high frequency (RF) plasma magnetron sputtering at different thicknesses. The PLA/WO_x_ film showed remarkable antibacterial properties against *E. coli*. The 50 nm and 100 nm thick coatings showed 99.9% reduction in oxygen permeation, and were found to be effective for sustainable active food packaging [89].

Wei et al. coated PLA film with alumina (Al_2_O_3_) using dielectric barrier discharge plasma-assisted atomic layer deposition, and the resultant film exhibited enhanced barrier and mechanical properties without diminishing the transparency or degradation rate. PLA filmmaking is suitable for food packaging applications [90]. Extruded PLA films were coated with a prepared PLA/SiO_2_ hybrid sol by Bang et al. Isocyanatopropyltriethoxysilane (IPTES) was employed as a silane coupling agent. Silica incorporation managed to decrease the gas permeation, and retained the transparency of the PLA film by up to 92% [91].

### 5.2. Macromolecular Colloidal Coatings

Enzymes, polyphenols, and biopolymers like polysaccharides, proteins, etc.,are the compounds that generally form macromolecular colloids, and have been reported successfully as coatings on packaging surfaces [92,93,94,95,96,97,98]. Carotenoids, alkaloids, phenolic acids, flavonoids, monoterpenes, isoflavones, and aldehydes are some of the active compounds found in plants that are being explored for active packaging [99]. Phenolic compounds are the most important active compounds among them [100]. The main principle for binding these bioactive compounds to a polymeric surface is adsorption via electrostatic interactions, ligand-receptor pairing, and covalent bonding. A prior functionalisation by treatment with ionised gas, UV irradiation, etc. is essential for polymeric films like PP, which does not have reactive side chain groups and are hydrophobic in nature [101]. There are many reports on macromolecular colloidal dispersions, especially polysaccharides such as chitosan, cellulose, xanthan, etc., as surface coatings for commercial plastic films [102,103,104].

To begin with, Fras Zemljič and group investigated the effect of chitosan bound to the surface of PET films. The macromolecular chitosan solution with a concentration of 1.5% (*w*/*w*) was prepared in bidistilled water, and the previously activated PET film was immersed in this solution and then dried. It was investigated that chitosan was bound successfully, due to its protonated amino groups, and a study was performed on the adsorption/desorption of chitosan on the film surface of the PET, which confirmed the reversible binding of chitosan on the surface of the PET. Also, the antibacterial studies proved the successful activity against *E. coli* and *L. monocytogenes* and fungi *C. albicans*, which are obvious causes of food spoilage [105]. Nguyen et al. used a different series of polysaccharides, nanocellulose and nanochitin, as coating on polypropylene films by layer-by-layer construction. The coating layers reduced the permeability to oxygen and water vapour significantly, and the resulting films were highly transparent, thermally recyclable, and prevented bacterial adhesion, making them an ideal candidate for food packaging. Many other polymers, such as pullulan, were also reported as coatings for various synthetic packaging materials [106].

Polyphenols are another important class of active agents employed as macromolecular coatings. Habib et al. utilised poly/ascorbic acid (ASA) (vitamin C) in colloidal form to coat LDPE surface by plasma-enhanced grafting. LDPE films functionalised by plasma irradiation were dipped in a 10% aqueous solution of ASA and grafted. The ASA was grafted covalently onto the LDPE surface. The grafting was confirmed by wettability, adhesion and morphology studies. The resulting films showed an inhibitory activity of over 80–90% against *S. aureus* [107]. Contini et al. prepared a coating of citrus extract on the surface of polyethylene terephthalate trays (PET). A comparative study was performed of the effectiveness of α-tocopherol and citrus extract (flavonoids) as a colloidal coating. Solutions of α-tocopherol or citrus extract were pumped into the nebuliser by a remotely-controlled syringe pump, and converted to an aerosol by helium flow through the nebuliser. The citrus extract proved to be a more effective antioxidant, and extended the shelf life of cooked turkey meat by reducing lipid oxidation, which was attributed to its higher surface roughness as measured by an optical profilometer [108]. A two-layer strategy was implemented on UV/ozone activated PP and PE films. The first layer comprised of a macromolecular chitosan solution, which enabled antimicrobial activity, and the second layer consisted of a nanodisperse network of polyphenol extracts (thyme, rosemary and cinnamon extracts) embedded in chitosan nanoparticles that possessed simultaneous antioxidant and antimicrobial properties. In addition, the approach improved the barrier properties of the films significantly [95]. Another experiment by the group tested the bilayer strategy of Glaser et al. and the additive effect of antimicrobial chitosan together with antioxidant resveratrol as an adsorbate for PP and PE films. The macromolecular chitosan solution was applied as the first layer to the plasma-activated PP and PE films, and chitosan nanoparticles with integrated resveratrol were applied as the second layer using the web printing method; the resulting films showed great potential for active packaging applications [94]. Sanja Potrč et al. went one step further and coated PE and PP films with two layers, namely, a macromolecular chitosan solution as the first layer and chitosan particles with embedded catechin or pomegranate extracts as the second layer. The main active ingredients in pomegranate extracts were polyphenolic compounds. The pure chitosan coating showed significantly lower antimicrobial activity compared to the synergistic effect of the colloidal chitosan-polyphenol formulation [92]. In addition, Elena Stoleru et al. coated PE films with a dual bioactive layer based on a colloidal formulation of antimicrobial chitosan and antioxidant vitamin E using the electrospray technique. The antibacterial/antioxidant layer was bound covalently by amide or carbamate bonds, using both 1-ethyl-3-[3-dimethylaminopropyl] carbodiimide hydrochloride and N-hydroxysuccinimide or carbonyldiimidazole as coupling agents. The resulting films retained the properties of the PE packaging film because the electrosprayed layer was very thin, so that it affected only the surface properties, and they proved successful against three different bacterial strains and exhibited excellent antioxidant properties [93].

Barbosa-Pereira et al. developed another sustainable coating. The high antioxidant activity of polyvinylpolypyrrolidone wash solution extract (PVPP-WS), a natural extract obtained from a brewery waste stream, was manipulated here. Phenolic compounds such as flavanols, hydroxycinnamic acids and hydroxybenzoic acids present in the extract of PVPP-WS are responsible for the antioxidant activity. The LDPE was coated with the natural extracts and this film was applied to beef samples. The ability of the natural extracts to scavenge free radicals was compared with that of synthetic antioxidants. The natural extracts exhibited higher activity than the synthetic antioxidant BHT, and reduced lipid oxidation by up to 80% compared to the control [46]. A coating of nanofibrillated cellulose (NC) and nisin was applied to cold plasma treated biaxially oriented polypropylene/low density polyethylene (BOPP/LDPE) films by Peng Lu and his group, which improved the barrier properties and antibacterial activity of the films. The NC was responsible for the improved oxygen barrier, and nisin imparted antimicrobial properties to the films without compromising the barrier properties [104].

There are some reports in the literature on PLA coated with antimicrobial agents, as interest in this area has increased in recent years. In order to achieve antimicrobial and antioxidant activity, several studies have been conducted to evaluate the effects of active ingredients such as polyphenols like thymol, eugenol and some natural extracts like cinnamon, garlic, clove, lemongrass, green tea, cumin, fennel oil, etc. on the thermal, optical, barrier, mechanical and biodegradability properties of the PLA. Most positive effects have been demonstrated on these properties [109,110,111,112,113].

Benbettaieb et al. studied the deposition of thin coatings of natural biopolymers (gelatine) in which bioactive agents were incorporated to develop active packaging materials while maintaining their biodegradability and food contact allowance. Two phenolic acids (tannic acid and gallic acid) were incorporated into macromolecular coatings of gelatine. These coated PLA films showed a reduction in moisture permeability and a slight change in the thermal properties of the PLA. The incorporation of the phenolic acids produced a controlled bioactive profile of the films [114].

In one of the interesting studies, inspired by the excellent adhesion and versatility of catechol groups based on shell bionics, a catechol functionalised layered clay (LDHs@ QUE -Cu) was firstly synthesised by adsorption and complexation of the natural active polyphenol quercetin (QUE) with copper ions on the surface of layered double hydroxides (LDHs). Active multilayer PLA composite films were prepared by integrating LDHs@ QUE -Cu (0.5–5 wt%) into chitosan (CS)/poly(vinyl alcohol) (PVA) coatings [115]. Moreover, the application of a zein coating loaded with quercetin at 5 wt% to an extruded PLA film changed its colour, but maintained its transparency and introduced bioactivity. The functional films thus produced exhibited the characteristic yellowish hue that resulted from the incorporation of zein and quercetin [116].

Strong antibacterial activity against *E. coli* and *Staphylococcus aureus* of PLA films, with and without specific nanoclays, impregnated additionally with thymol or cinnamon extracts by scCO_2_ impregnation, was obtained by dynamic contact tests. Thymol and cinnamon extracts produced excellent inhibition of both bacteria [113].

The antibacterial activity of PLA films integrated with cinnamon against *E. coli* and *Listeria innocua* was studied using the disk diffusion method for 13 days. A bacteriostatic effect against *E. coli* was observed in the first 6 days, followed by a bactericidal effect. However, the growth of L. *innocua* was inhibited for only 9 days, which may be attributed to the progressive evaporation of the cinnamon during the test period [117].

PLA films were modified by coatings containing beta-cyclodextrin inclusion complexes with thymol and carvacrol (at 1.5, 2.5, and 5 wt%). The bioactivity was evaluated for 10-day inhibition of the fungus *Alternaria alternata* using the vapour phase diffusion method. Mould growth was completely inhibited by these PLA films, with the highest concentrations of phenols found in inclusion complexes [118].

A polylactic acid film was coated with chitosan or a chitosan/sodium caseinate mixture enriched with rosemary essential oil. The results of the in vivo test showed that the active films were able to reduce the oxidation of the meat during storage under anaerobic conditions in a modified atmosphere, i.e., reduce the malondialdehyde concentration of the chicken meat by 50% [119].

Antipack ^TM^, manufactured by Handary in Belgium, is an example of a commercial PLA antimicrobial packaging product, which is a film made of a PLA/starch-based material containing an antifungal agent. This product is designed to retard the growth of yeast and mould during shelf life by delivering chitosan, including natamycin, to the surface of solid foods such as cheese, fruit, vegetables, meat and poultry [120].

Pure Chitosan coatings have been used to modify PLA films to introduce the active concept. (PLA) films were coated with squid chitosan in different amounts (0, 1, 3 and 5 phr) using the cast coating method. The objective of the work was to investigate whether this coating acts as an antimicrobial agent, and has the potential to extend the life of vegetables and fruits packaged with these functional polylactic acid films. When perishability was tested, it was found that the food was not deteriorated by microbial spoilage, but all vegetables and fruits with a higher chitosan concentration on the PLA film were more shrivelled and wilted, which made it unacceptable for selling. The possible explanation was that the residual acetic acid after cast coating caused hydrolytic degradation of the PLA, and the resultant by-products led to the shrivelling of vegetables and fruits. Hence, the coating was proven successful against bacterial activity, while the coating method was unsuccessful [121].

Interestingly, active coatings containing modified and unmodified polymeric chitin-lignin nanoparticle complexes as complex colloidal systems were prepared and applied to extruded PLA-based sheets. The prepared coating was applied to extruded PLA-based sheets using a brush technique. The studied effects on the mechanical and thermal properties were lower, and the uncoated and coated films exhibited similar properties, regardless of which active ingredient they contained [122].

Seok-Hoon Park et al. demonstrated a significant improvement in the barrier properties of poly(lactic acid) films coated with chitosan or chitosan/clay nanocomposite by checking the oxygen and water vapour permeability [123].

Turalija et al. investigated antimicrobial modifications of biodegradable polylactide (PLA) using environmentally friendly antimicrobial agents (silver and chitosan combinations) and bio-based alcohols (glycerol and polyethylene glycol). The modified PLA films were particularly effective against *Staphylococcus aureus* and *E.coli,* and therefore could be used for food packaging in the future. The antimicrobial films made from biodegradable PLA offer a sustainable solution for the food packaging industry to extend the shelf life of certain foods [124].

Apicella et al. published a study on biodegradable films based on poly(lactic acid) coatings with natural olive wastewater extracts, including biopolymers for active food packaging. It was found that the antioxidant activity increased by up to 20% with increasing the concentration of olive wastewater extracts. Preliminary shelf-life tests confirmed the prospects of using these films as a 100% green alternative for preserving O_2_-sensitive foods with high respiration rates, such as fresh-cut avocados [125].

Jin and others investigated the efficacy of PLA/pectin films coated with nisin against *L. monocytogenes*. They reported that PLA/pectin and PLA films coated with nisin (1%, *w*/*v*) differed significantly in terms of their antimicrobial activity in the agar diffusion assay [126].

### 5.3. Associated Colloidal Coatings

Molecules such as polar lipids and surfactants and those with amphiphilic character, i.e., with a large solubility difference between their hydrophilic and hydrophobic segments, can assemble into association colloids, which include micelles, liquid crystals, microemulsions, and so on. This self-assembling property of molecules can be used effectively for the purpose of carrying active molecules, surface modification, or as colloidal dispersants. This can be beneficial in the food packaging industry, as they can be used as delivery systems for active ingredients [76,97].

Some of the active ingredients, especially plant-derived phenolic compounds which are known for their functional properties, such as antioxidant, anticarcinogenic, and antimicrobial activities, are not used effectively in packaging, due to their thermal instability and sensitivity to light, pH, oxygen, and other food components. Encapsulation of these compounds using this colloidal approach could rectify these issues to some extent and enhance their functional properties. Encapsulation of polymeric nanoparticles protects the active compounds from degradation, improves their solubility, and enables controlled release [127].

The techniques used for nanocapsulation are classified as particle size reduction, self-assembly, and solvent diffusion methods, based on their mechanisms for nanostructure formation. The main particle size reduction methods are high pressure homogenisation, ultrasonication, and microfluidisation. Self-assembly methods include micelle/liposome formation, spontaneous emulsification and complexation. Solvent diffusion methods include the emulsion diffusion method, nanoprecipitation, etc. The emulsion diffusion method is a commonly used method in which a regular oil-in-water emulsion containing the biopolymer in the dispersed phase (biopolymer, oil and organic solvent) is prepared in the first step, using conventional emulsification methods such as high-speed homogenisation equipment; water is then added, resulting in the diffusion of an organic solvent into the continuous phase (water), causing the separation of the biopolymer and oil, leading to the formation of encapsulated nanoparticles [128].

In an approach by Pereira et al., phenolic compounds (mainly ascorbic acid) extracted from guabiroba fruit were encapsulated using poly(d,l-lactic acid-co-glycolic acid) nanoparticles (PLGA) by emulsion evaporation. The PLGA nanoparticles proved to be an effective delivery system for phenolic compounds due to their improved functional properties, even at lower amounts than originally required [127]. Liposomes are types of associated colloids containing vesicles composed of one or more bilayer membranes, and have gained importance due to their ability to encapsulate antimicrobial agents, aroma components, and enzymes. Because they have a nonpolar lipid and a polar aqueous phase, liposomes can encapsulate both polar and nonpolar compounds. For example, Makwana et al. encapsulated cinnamaldehyde with lipid bilayers of polydiacetylene-N-hydroxysuccinimide (PDA-NHS) to form nanoliposomes, and immobilised them on PLA films [129]. Similarly, a natural phenylpropanoid dimer, curcumin, was entrapped by Navneet Dogra et al. in liposome-like polydiacetylene/hospholipid nanovesicles supplemented with N-hydroxysuccinimide and glucose, and these nanovesicles were attached covalently to silanised glass for food packaging applications. The nanoparticles functionalised with curcumin exhibited bactericidal activity against both gram-negative (*E*. *coli*) and vegetative cells of gram-positive (*B. cereus*) bacteria [130].

Elena Stoleru and her group demonstrated the immobilisation of clove essential oil and argan oil stabilised in emulsions with chitosan on PLA activated by plasma treatment. The resulting films showed low permeability to oxygen, high radical scavenging activity, and strong growth inhibition *for Listeria monocytogenes, Salmonella Typhimurium*, and *E. coli* bacteria [131].

A summary of active coatings as colloidal formulation on PLA films is given in Table 2.

## 6. Antiviral Approach through Surface Coatings as Essential Strategy

**Figure 13:**
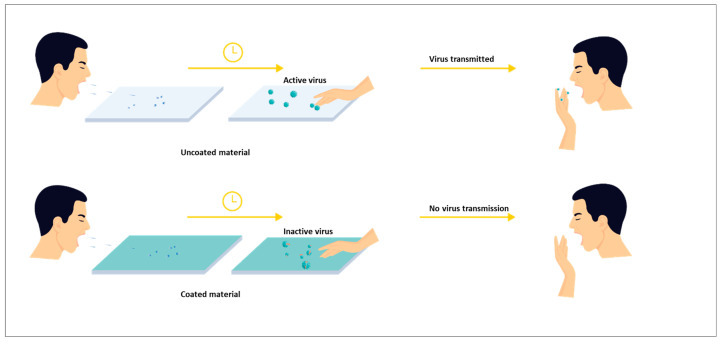
Schematic representation of the antiviral concept through surface coating.

Potential alternatives to improve food safety and quality against foodborne infections are antiviral coatings and packaging solutions. Viral disease outbreaks are caused mostly by the contamination of food, especially meat, vegetables and fruits, with viral pathogens. In addition, packaging materials are thought to contribute significantly to cross-contamination, the indirect transmission of viral infections. Given that SARS-CoV-2 is able to persist on the surfaces of packaging materials throughout the whole period between production and consumption, this long stability of the virus raises serious concerns and risks for the packaged food trade [132]. Thereby, the production of antiviral materials such as active coatings, films and multilayer packaging systems, has seen an increase in the research arena. The schematic representation of the antiviral concept through surface coating can be seen in Figure 13. The application of antiviral agents in colloidal solutions on the surface of food packaging is described in more detail in another section [133]. Nanoscale colloidal formulations have helped to solve the problems of solubility and bioavailability of antiviral agents, and have provided the agents with unique properties that increase their efficacy and selectivity toward virions [134]. Metal and metal oxide nanoparticles are the most studied class of antiviral nanoparticles that form multimolecular colloidal systems [135]. Zinc oxide nanoparticles have been studied extensively by various researchers and show antiviral activity [136,137].

Huy et al. studied the antiviral activity and cytotoxicity of silver nanoparticles and found that they are not toxic to cell cultures at concentrations of 100 ppm or less, but are toxic to non-enveloped viruses. The results demonstrated that these metal nanoparticles can be used as coatings for packaging materials. Bao et al. reported polylactic acid oligomers (PLA) as biologically safe and environmentally friendly functional antimicrobial agents against pathogens such as viruses (H1N1, H3N2, and SARS-CoV-2), bacteria (*E. coli, S. aureus, K. pneumoniae, MRSA*), and fungi (*C. albicans*) [138].

However, polymers with long chains and branches forming the macromolecular colloids have been shown to have high antiviral capacity compared to monocompounds. Recently, a variety of polymers have been developed to meet the requirements of ubiquitous bacterial and viral pathogens [139]. Nowadays, more research is being conducted on ionic polymers, including polysaccharides such as alginate, agar, carrageenan, chitosan, polylysine, and polyarginine [140]. Essential oils and plant extracts have also recently been investigated for their antiviral properties [141]. The antiviral activity of essential oils and natural extracts is due to the bioactive compounds, usually polyphenols, they contain. Many essential oils were found to be quite successful in containing the new coronavirus strain SARS-CoV-2 [142]. Mizieliska et al. used nanoparticles of ZnO, carvacrol, and geraniol to create an external coating that could be effective against viruses such as SARS-Co-V2. According to the study, coatings with a higher concentration of geraniol or carvacrol and very little nano-ZnO were effective against both Gram-positive and Gram-negative bacteria. Also significant is the observation of a synergistic interaction between these active ingredients. Thus, it was found to be the best packaging material to increase the quality and freshness of food. Due to its antiviral properties, it can also be coated externally. The coatings had a moderate effect on Phi 6 phage, a representative of viruses such as coronaviruses [143]. Fabra et al. studied the virucidal activity of cinnamaldehyde on virus suspensions at two different temperatures and contact times. The antiviral activity against norovirus surrogates improved with increasing the cinnamaldehyde concentration at 37 °C for 2 h, increasing its efficacy for final application in antiviral food packaging or contact surfaces [144].

Biopolymers can be employed as carrier matrices for active antiviral components. Also, there are biopolymers like heparin which possess antiviral characteristics of their own [140]. Depending on the storage parameters, such as relative humidity, temperature and pH, the active components can be released from the polymeric materials using the appropriate colloid formulation. Plohl et al. have reported the potential of chitosan-based coatings to be used as antiviral coatings on packaging surfaces [145]. Sukano ^®^ has developed an antiviral masterbatch for PLA applications that helps in surface removal of viruses and other bacteria. Sukano’s antiviral masterbatches work by using a patented formulation and technology to incorporate an antiviral ingredient directly into the polymer. This creates a strong and scientifically validated safety layer that prevents the transmission of viruses to product surfaces [146]. Abdulkareem et al. modified the PLA surface by plasma treatment and grafting ascorbic acid and fumaric acid. The resulting film was found to be antiviral, and suitable for food packaging [147]. The development of antimicrobial PLA films with improved physical and mechanical properties and antimicrobial activity remains a challenge, due to the inherent brittleness and hydrophobicity of this polymer. A major challenge is also multifunctionality, i.e., maintaining multiple functions together so that they are not contradictory. Some of these material limitations can be overcome by using current and advanced technologies, such as colloidal technology and chemistry, including nanotechnology.

## 7. Challenges and Future Trends

In Colloidal chemistry it is important to understand in detail the physicochemical and bioactive properties of the surface, as these interface with the food, and, thus, the surface parameters are the driving force for the effectiveness of the packaging in contact with the food. To date, more emphasis has been placed on the mechanical and structural properties of PLA and less on this topic, so there is still room for research here.

An important consideration in the development of coatings for active packaging applications is the consideration of toxicity and potential for regulatory approval, not only of the active ingredient, but also of any additives or crosslinkers that may be used. It is extremely important that natural, biodegradable and non-toxic compounds such as biopolymers and polyhenols are the focus in coatings. In addition to safety, opportunities for new products are seen in material costs, improved safety and waste reduction. When these priority compounds are in a colloidal structure less substance is needed, while the colloidal structure ensures a large surface area and can act in a very efficient and controlled manner. When we consider the homogeneity and specific thickness required for coating strategies, colloidal macromolecular solutions such as biopolymers solutions, which can provide homogeneous and thick films, are at the top of the list.

In addition, the main challenge is to develop technologies that can reduce the amount of active substances to maintain adequate activity and keep migration levels below regulatory limits. In this context, a future direction to solve this problem could be the use of green immobilisation, such as enzymatic grafting and/or the development of synergistic effects of different natural compounds, such as colloidal formulations, which could allow reducing the required concentration.

However, further research is needed to develop more resilient and marketable biodegradable smart and active packaging materials. Although the results until now provided very useful insights into the production and application of biobased packaging materials for the food industry by explaining the properties of different bio-derived materials (starch, cellulose, chitosan, proteins, PLA) in terms of food packaging, environmental impact, life cycle assessment (LCA) and market aspects, they demonstrate clearly the potential of active biodegradable packaging that has been achieved under laboratory conditions but not yet translated effectively into practice. The risks associated with scale-up have hindered the demonstration of promising research results at a real scale, as they could not be combined into an integral system suitable for mass production. The limitations for biodegradable active packaging are also related to the following factors:

- Lack of knowledge about the material itself, its interactions, and its compatibility with existing packaging technology.
- The final performance of the packaging compared to its environmental and financial costs
- Incorporation of the new food packaging systems into integrated food chain concepts.
- The stability and safety of the new biodegradable active packaging: Materials have functional limitations.
- Effective industrial composting facility programmes–the latest need to support the concept of biodegradable packaging.
- Regulatory hurdles: Governments need to take appropriate support measures.

The implementation of sustainable and multi-active food packaging can, thus, only be improved by promoting the implementation of novel fundamental research, as well as novel technologies that are already close to practical application and meet the specific requirements of target foods. This can be done through knowledge transfer from universities and research institutes joining together to form an efficient consortium that will transfer relevant knowledge to food companies (preferably small and medium-sized enterprises (SMEs), and global companies) that can test the possibilities of the new technologies for their specific products in feasibility studies.

Finally, with the recent outbreak of SARS-CoV-2, the demand for antiviral coatings has become inevitable [143]. Since the virus remains stable on surfaces for several days and causes cross-infections, research is underway for a coating for the likely surfaces where the virus can spread. However, experiments with viruses require sophisticated safety measures, which are expensive because they are harmful to humans, so they can only be performed in a few laboratories. Therefore, finding bacteriophages that serve as good surrogates is a challenging task. Although there are antiviral biopolymers that are potential candidates for antiviral coatings for food packaging, their spectrum of activity is very low and they are relatively less stable. Hence, an improvement is required on these disadvantages. Biopolymers or biopolymers combined with other materials are required that target for a wider range of viruses [148].

## Figures and Tables

**Table 1:** Classification, mode of action and some examples of active agents.

Active Agents					
Releasing System			Non-Releasing System		
Type	Activity	Example	Type	Activity	Example
Anti-microbials	antimicrobial activity	chitosan, nisin, lysozyme, [52] titanium dioxide (TiO_2_) and zinc oxide (ZnO), Silver (Ag) [53]	Oxygen scavengers	absorb Oxygen/products of primary oxidation	Curcumin [54] gallic acid, iron, palladium [13]
Antioxidants	Free radicals and peroxides react to retard or block the actual oxidation reactions.	Tocopherol, carvacrol, quercetin, catechin, thymol [55] silver, copper, TiO_2_ ZnO [56]	Ethylene scavengers	scavenge ethylene by adsorption process and cation exchange.	KMnO_4_, halloysite nanotubes (HNTs), zeolites [57], titanium dioxide [12]
Carbon dioxide emitters	Produce carbon dioxide	Citric acid, sodium bicarbonate [58]	Carbon dioxide scavengers	Absorb CO_2_ from the package headspace	calcium hydroxide, Na_2_CO_3_, Mg(OH)_2_ [59]
Flavour emitters	Substances able to release flavours	Allyl isothiocyanate, Citral, Ethyl hexanoate [60]	Moisture scavengers	able to absorb, adsorb H_2_O	fructose, cellulose, NaCl, calcium hydroxide, zeolites, Montmorillonite, halloysite nanotubes [14]

**Table 2:** Some of the coatings applied on PLA films for active packaging.

Compounds Used	Colloidal System	Type of Deposition	Physico-Chemical Properties	Ref.
Aluminium-doped zinc oxide	Multimolecular	PVD-magnetron sputtering	Antibacterial properties UV-blocking properties	[87].
Tungsten oxide	Multimolecular	PVD-plasma magnetron sputtering	Antibacterial properties Oxygen barrier	[89]
Alumina	Multimolecular	CVD-dielectric barrier discharge plasma-assisted atomic layer deposition	Water vapour barrier property Increase in tensile strength	[90]
Gelatine-tannic/ gallic acid	Macromolecular	Cast coating-With thin-layer chromatography spreader	Water vapour barrier property Antioxidant property Antibacterial properties	[131]
Zein coating loaded with quercetin and Cellulose nanocrystals	Macromolecular	Screen printing	Lower Young’s modulus Higher elongation values antioxidant	[116]
Nisin	Macromolecular	Diffusion coating	Antibacterial properties	[126]
Nano encapsulated cinnamaldehyde	Associated colloids	Dip coating	Antibacterial properties	[129]
Curcumin polydiacetylene/phospholipid- complex	Associated colloids	-	Controlled release of antibacterial agents	[130]

## Data Availability

Not applicable.
